# Impressive Response to Concomitant Platinum-based Chemotherapy and Yttrium-90 in a Patient with Heavily Pretreated Triple-negative Breast Cancer Widely Metastasized to the Liver

**DOI:** 10.7759/cureus.1402

**Published:** 2017-06-28

**Authors:** Aurelio Castrellon, Steven M Nguyen, Federico Bengoa, Ana Botero, Luis E Raez

**Affiliations:** 1 Breast Cancer Center, Memorial Cancer Institute; 2 Department of Civil Engineering, Carnegie Mellon University; 3 Department of Interventional Radiology, Memorial Healthcare System; 4 Department of Radiation Oncology, Memorial Healthcare System; 5 Medical Oncology and Hematology, Memorial Cancer Institute

**Keywords:** metastatic breast cancer, yttrium-90, chemotherapy, triple-negative breast cancer

## Abstract

Metastatic triple-negative breast cancer (TNBC) constitutes a heterogeneous group of diseases with systemic treatment options limited to cytotoxic chemotherapy at the time being. The disease tends to affect visceral organs more frequently when compared to hormone receptor-positive breast cancer. The prognoses of patients with heavily pretreated disease affecting the liver are very dismal. We present the response to radioembolization and systemic chemotherapy in a seriously ill patient who had undergone previous lines of chemotherapy for TNBC with extensive liver metastases.

## Introduction

Triple-negative breast cancer (TNBC) accounts for approximately 20% of breast cancers (BC) diagnosed worldwide, representing almost 200,000 cases each year [1]. TNBC is characterized by the absence of expression of estrogen receptors (ER) and progesterone receptors (PR) in addition to the lack of amplification of the human epidermal growth factor receptor 2 (HER2)/Neu gene [2]. Unlike hormone receptor-positive (HR+) and HER2 overexpressing breast cancers, TNBC is unresponsive to endocrine therapy and HER2-targeted agents, which limits treatment options to conventional cytotoxic chemotherapy [3]. Patients with metastatic disease typically experience rapid progression through several lines of chemotherapy, and overall survival (OS) in the metastatic setting is usually poor with reports ranging from nine to 13 months [4].

Selective internal radiation therapy (SIRT) has arisen in the past few years as a very useful therapeutic option in select patients with unresectable cancers or in those who cannot be treated surgically, such as with hepatocellular carcinoma (HCC) or liver metastases [5]. Yttrium-90 (Y-90) radioembolization involves the administration of microspheres by catheterization of the hepatic artery. Patients who are candidates for radioembolization include those with unresectable primary liver cancer, metastatic colorectal cancer, and neuroendocrine tumors. The treatment requires patient-individualized planning with cross-sectional imaging and arteriograms. Contrast computed tomography (CT) and/or contrast-enhanced magnetic resonance imaging (MRI) of the liver is required to assess the tumor and normal liver volumes, portal vein status, and extrahepatic tumor burden. The maximum response in terms of size reduction for HCC could take up to four to six months [6].

There is limited data assessing the effect of SIRT on liver metastases from metastatic breast cancer (MBC). The earliest study performed by Bangash and colleagues in 2007 demonstrated positive tumor response on 18F-fluorodeoxyglucose-positron emission tomography (18F-FDG PET) in 63% of patients. There was a trend toward better overall survival (OS) in the patients experiencing over 25% decrease in the size of the tumor burden [7]. The efficacy and safety of combining systemic chemotherapy with SIRT have only been evaluated in colorectal cancer. The SIRFLOX study randomized 530 patients to receive FOLFOX chemotherapy (5-fluorouracil, leucovorin, and oxaliplatin) ± bevacizumab (Avastin®; Genentec, Inc., San Francisco, CA) with SIRT using Y-90 resin microspheres as the first-line treatment for liver metastases from metastatic colorectal carcinoma. In this study, the addition of SIRT increased the median progression-free survival in the liver from 12.6 months to 20.5 months (P = 0.002). Grade ≥ 3 adverse events, which included nausea, pain, and liver dysfunction, were reported in 73.4% and 85.4% of patients for control vs. SIRT [8].

We present the response to platinum-based chemotherapy with concomitant Y-90 therapy in a patient with heavily pretreated, widely metastatic TNBC to the liver, who experienced severe deterioration of performance status and liver function tests prior to starting treatment.

## Case presentation

The patient’s timeline of disease progression and treatment is outlined in Table 1. In November 2014, a 62-year-old woman without significant family history for breast or ovarian cancer presented with a 3 cm left breast mass and axillary adenopathy. Ultrasound-guided biopsy of the breast mass and axillary lymph node reported ER-/PR-/HER2 Neu negative invasive ductal carcinoma. The patient was sent for staging studies, which included a CT of the chest, abdomen, and pelvis. Her CT revealed a left axillary metastatic adenopathy, 3.3-cm hypoattenuating mass at the junction of segments 2 and 3, and a 0.4-cm hypoattenuating lesion in segment 8 of the liver. Biopsy of the liver lesion revealed metastatic carcinoma consistent primary breast cancer that was ER/PR negative and HER2/Neu negative.

**Table 1:** Treatment Timeline

Date	Disease Progression	Treatment
January 2014	Diagnosed with Stage IV breast cancer	Capecitabine, 1000 mg/m2, days 1-14 Q 21 d x 5 cycles
July 2015	Increased size of mass in the left lobe of the liver measuring 6 cm compared to 3.3 cm previously. No other liver lesions.	Nab-paclitaxel, 100 mg/m2, days 1, 8, 15 Q 28 d x 6 cycles
January 2016	Increased size of left lateral segment of the liver since the previous exam, measuring 8.1 cm	Gemcitabine, 1250 mg/m2, day 1, day 8 Q 21 d x 3 cycles
April 2016	Marked multifocal bilobar hepatic metastatic disease, mainly localized to the left lobe liver segments 2 and 3. New involvement of left lobe segments 4A, 4B, 5, and 7.	40.5 mCi Yttrium-90 SIR given concomitant with Paclitaxel, 80 mg/m2, + Carboplatin AUC 2 weekly followed by a second course of Y-90 a month later

**Figure 1:**
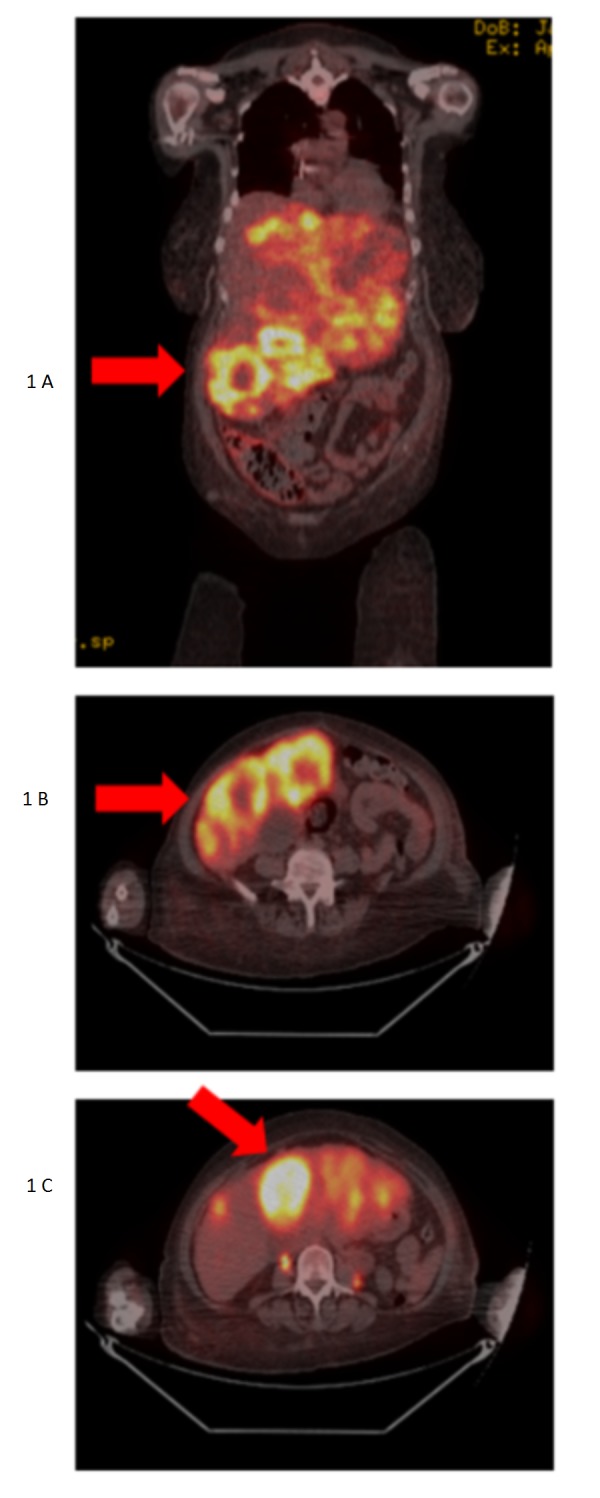
Fluorodeoxyglucose-positron emission tomography/computed tomography - April 2016 Fluorodeoxyglucose-positron emission tomography/computed tomography demonstrating multifocal bilateral high-grade hypermetabolic bilobar hepatic metastases. Involvement is most severe in the region of the left and inferior right liver lobe. Confluent disease cannot be measured accurately due to blending of adjacent tumor.

Because of hair loss concerns, she initiated therapy with capecitabine (Xeloda®; Hoffmann-La Roche, Inc.) at the dose of 1,000 mg/m2 BID (days 1 to 14 of a 21-day cycle). Her last dose of capecitabine was given on July 15, 2015, and it was discontinued due to progression in the liver. Second-line chemotherapy for Stage IV TNBC consisted nab-paclitaxel (Abraxane®; Celgene Corporation, Summit, NJ) 100 mg/m2 weekly (day 1, day 8, and day 15 of a 28-day cycle). The patient completed 6 cycles of chemotherapy. Treatment was discontinued secondary to further size progression of the liver lesions in late January 2016. Third-line chemotherapy with gemcitabine (Gemzar®; Eli Lily and Company, Indianapolis, IN) 1,250 mg/m2 (days 1 and 8 of a 21-day cycle) was given from February 2016 to May 2016 and was discontinued secondary to uncontrolled liver metastases. The 18F-fluorodeoxyglucose-positron emission tomography-computed tomography (FDG-PET/CT) demonstrated multifocal high-grade hypermetabolic bilobar hepatic metastases (Figure 1 A-C). At this point, her prognoses was dismal. The patient had developed significantly worsening performance status and was suffering from uncontrolled pain. End-of-life care discussion took place with family members. She had recently transferred care to our institution. The patient was presented to interventional radiology for Y-90 therapy. On April 2016, 40.5 mCi Yttrium-90 SIR-Spheres were administered selectively via the hepatic artery.

**Figure 2:**
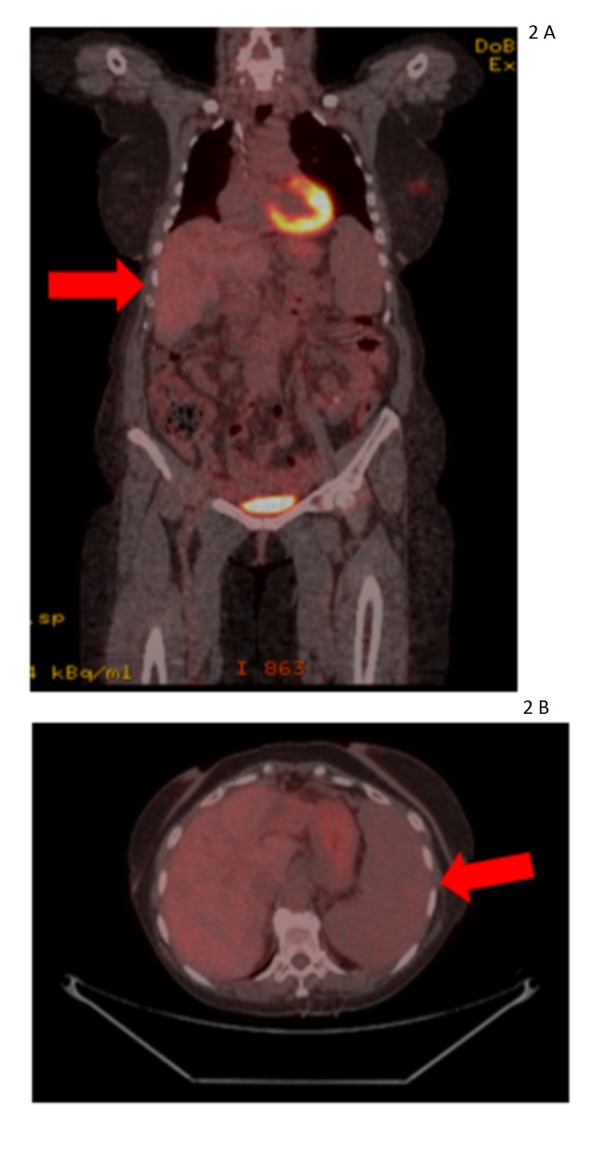
Fluorodeoxyglucose-positron emission tomography/computed tomography - April 2017 Fluorodeoxyglucose-positron emission tomography/computed tomography demonstrating marked improvement of hepatomegaly with no residual active hepatic metastases. New splenomegaly is seen.

Concomitantly with Y-90 SIRT, she was started on weekly paclitaxel, 90 mg/m2, combined with carboplatin AUC 2. She started to show clinical signs of improvement with a marked decrease in hepatomegaly on physical exam. On May 2016, a second Y-90 procedure was performed. She remained on chemotherapy until September 2016. Treatment was stopped secondary to an allergic reaction to carboplatin. There was consideration for enrolling her in the Medivation 3800-13 Phase 2 Single-arm Open-label Clinical Trial of Extended Treatment Safety in Patients Treated with Talazoparib (NCT02921919), but the patient was unable to participate due to persistent thrombocytopenia (platelet count < 70,000/uL). We opted to follow her clinically and perhaps start chemotherapy with any sign of progression. Currently, she has not received any therapy since September 2016. Subsequent PET/CT scans have demonstrated progressive size decrease in her liver lesions. Her most recent PET/CT scan on April 2017 (Figure 2 A-B) indicates improved hepatomegaly, no definite residual active hepatic metastasis, a recurrent hypermetabolic left breast mass, a new L2 osseous lesion, and new splenomegaly. Further palliative chemotherapy and genetic testing to determine potential treatment with poly ADP ribose polymerase (PARP) inhibitors are planned.

## Discussion

The case presented in this report illustrates an impressive response to systemic chemotherapy for TNBC given concomitantly with Y-90 in a patient who was severely ill with a poor prognosis. She has survived one year since treatment with an exceptional quality of life. We were unable to offer further therapy when she developed an allergic reaction to carboplatin because of her persistent thrombocytopenia. The patient has developed splenomegaly, which is believed to be associated with hepatic fibrosis and/or portal hypertension post-radioembolization with Y-90 [9]. A retrospective analysis demonstrated splenomegaly following radioembolization, which could be associated with a low platelet count; however, no significant bleeding diathesis has been reported [10]. We believe that the concomitant use of chemotherapy and Y-90 should be considered in cases similar to this one.

## Conclusions

Y-90 therapy, in combination with platinum-based chemotherapy, showed a significant response in the case of a patient with advanced metastatic breast cancer to the liver. The patient has survived over one year after being in a terminal condition. We are planning further palliative therapy strategies.
